# Paeonol Ameliorates Ulcerative Colitis in Mice by Modulating the Gut Microbiota and Metabolites

**DOI:** 10.3390/metabo12100956

**Published:** 2022-10-08

**Authors:** Jiahui Zheng, Huan Li, Pei Zhang, Shijun Yue, Bingtao Zhai, Junbo Zou, Jiangxue Cheng, Chongbo Zhao, Dongyan Guo, Jing Wang

**Affiliations:** 1 State Key Laboratory of Research & Development of Characteristic Qin Medicine Resources (Cultivation), Shaanxi University of Chinese Medicine, Xi’an 712046, China; 2 Shaanxi Key Laboratory of Traditional Chinese Medicine Foundation and New Drug Research, Shaanxi University of Chinese Medicine, Xi’an 712046, China; 3 State Key Laboratory of Natural Medicine, China Pharmaceutical University, Nanjing 210009, China; 4 Key Laboratory of Shaanxi Administration of Traditional Chinese Medicine for TCM Compatibility, Shaanxi University of Chinese Medicine, Xi’an 712046, China

**Keywords:** paeonol, ulcerative colitis, dextran sodium sulfate, gut microbiota, metabolomics

## Abstract

Ulcerative colitis (UC) is a chronic recurrent inflammatory disease of the gastrointestinal tract. Recent studies demonstrate that the phenolic tannin paeonol (Pae) attenuates UC in mouse models by downregulating inflammatory factors. However, its molecular mechanism for UC treatment has not been explored from the perspective of the gut microbiota and metabolomics. In this study, we investigated the effects of Pae on colonic inflammation, intestinal microbiota and fecal metabolites in 3% dextran sodium sulfate (DSS) induced BALB/c UC mice. Pae significantly improved the clinical index, relieved colonic damage, reduced cytokine levels, and restored the integrity of the intestinal epithelial barrier in UC mice. In addition, Pae increased the abundance of gut microbiota, partially reversed the disturbance of intestinal biota composition, including Lactobacillus and Bacteroides, and regulated metabolite levels, such as bile acid (BA) and short-chain fatty acid (SCFA). In conclusion, our study provides new insight on Pae remission of UC.

## 1. Introduction

Inflammatory bowel disease is characterized by uncontrollable, non-specific, chronic immune-mediated inflammation of the intestine and can be classified as Crohn’s disease or ulcerative colitis (UC). The main lesions of UC in the mucosa and submucosa of the colon and rectum present as continuous open ulcers, with common clinical signs of gastroenteritis, fever, diarrhea, rectal bleeding, and fecal mucus [1,2,3,4]. Epidemiological studies suggest that the incidence of UC is increasing every year, and it has been classified by the World Health Organization as a “modern intractable disease” [5,6,7]. Nevertheless, the pathogenesis of UC is not entirely clear. It is generally accepted that UC is associated with intestinal mucosal damage, immune dysfunction, genetic susceptibility and microbial dysregulation [8,9]. In this regard, an increase in inflammatory cytokines is thought to be an important trigger for the development and progression of UC [10]. In addition, numerous studies have shown that UC is a result of abnormal changes in the gut microbiota and metabolites, such as bile acids (BAs), short-chain fatty acids and tryptophan, which initiate disruption of the intestinal barrier, leading to increased paracellular permeability, reduced colonic tight junction (TJ) protein integrity and an increased inflammatory response [11,12,13,14,15]. Interestingly, BAs act as pleiotropic signaling metabolites that are involved in the UC developmental process through dynamic interactions with the intestinal microbiota [11,16]. Some studies have pointed out that BAs are FXR receptor agonists and that FXR induces small heterodimer partner (SHP) in the liver and FGF19 (FGF15 in rodents) that bind with liver fibroblast growth factor (FGF) receptor 4 (FGFR4) via an endocrine mode after secretion from the intestine; both signal to inhibit CYP7A1, the rate controlling enzyme in the de novo synthesis of bile acids [17,18,19,20], and restore bile acid homeostasis in models of colitis, thus delaying the pathological progression of colitis [21]. Therefore, pathways related to the regulation of the intestinal flora and metabolism are potential targets for UC prevention and treatment.

Current clinical treatments for UC include steroids, aminosalicylates, immunosuppressive agents and biologics. However, interventions with these drugs are frequently associated with side effects, leading to a deterioration in the quality of life that drives many UC patients to seek alternative and/or complementary therapies [22,23]. Based on their general safety and presumed efficacy since ancient times, natural plants rich in bioactive components have been considered as promising alternative options for the treatment of these intestinal disorders.

Phenolic compounds, widely found in plants, are important natural antioxidants that can improve human health. Polyphenols may improve gut health via their anti-inflammatory and antioxidant activities, which are associated with protection of the intestinal barrier, modulation of immune function, increase in the relative abundance of beneficial bacteria and inhibition of pathogenic bacteria [24,25]. Pae (Paeonol, 2’-hydroxy-4’-methoxyacetophenone; C_9_H_10_O_3_) is a naturally occurring phenolic compound in peony bark with a variety of biological effects [26,27], including anti-inflammatory [28], analgesic [29], neuroprotective and anti-atherosclerosis effects [30,31]. Several studies have reported that Pae’s anti-inflammatory effects may alleviate UC. Zong et al. [32] demonstrated that Pae improves TNBS-induced UC by modulating pro/anti-inflammatory cytokine levels. In addition, Ge et al. [33] showed that Pae improves dextran sodium sulfate (DSS)-induced UC by reducing inflammatory damage and inhibiting pathogenic bacteria. However, these studies are limited, focusing mainly on whether Pae intervention is protective against inflammation. Given the contribution of dysbiosis and metabolic disturbances in UC, the precise role of Pae in UC, and in particular its effect on pathways related to gut microbiota and metabolic regulation, needs further investigation.

The DSS-induced mouse model of UC behaves similarly to human UC and could serve as a beneficial approach to study UC pathogenesis and pharmacological treatments [34,35,36]. Therefore, in this study, a 3% DSS solution was used to induce UC in mice and to investigate the protective effect of Pae. We employed 16S rRNA sequencing and ultra-performance liquid chromatography/tandem mass spectrometry (UPLC-MS/MS) to further analyze the potential mechanisms of action of Pae in ameliorating UC. In addition, we also explored the effects of Pae on BAs-related pathways. The results provide a scientific basis for further expansion of the clinical application of Pae in treating ulcerative diseases.

## 2. Materials and Methods

### 2.1. Reagents

**Figure 1:**
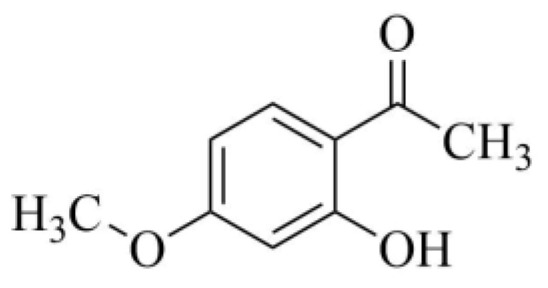
Paeonol chemical structure.

Paeonol (Pae, MedChemexpress, HY-N0159, Figure 1), dextran sulfate sodium (DSS, 36,000–50,000 kDa; MP Biomedical, California, USA), and Salazosulfapyridine (SASP, Lot No. 09190605, Shanghai Xinyi Tianping Pharmaceutical Co., Ltd.) were stored as recommended by the manufacturers and used at the indicated concentrations.

### 2.2. Animal Experiments and Sample Collection

A total of 40 male BALB/c mice of SPF grade, (6-8 weeks old, weight 20~22 g) were purchased from Chengdu Dashuo Co. The mice were housed under standard conditions (temperature 25 ± 2 °C, relative humidity 40 ± 5%, light and dark cycles 12/12 h, free access to water and standard diet) throughout the experimental period. Animal experiments were reviewed and approved by the Experimental Animal Ethics Committee of Shaanxi University of Chinese Medicine.

After 7 days of adaptive feeding, the mice were randomly divided into 5 groups (Control, DSS, DSS+ Pae-L, DSS+ Pae-H and DSS+SASP; *n* = 8/group). The Control group was given 0.5% CMC-Na solution and the other groups were treated with 3% DSS (wt./vol, dissolved in distilled water and administered ad libitum) for 7 consecutive days to induce UC. While modeling, the treatment groups were administered low-dose Pae (Pae-L, 50 mg/kg), high-dose Pae (Pae-H, 100 mg/kg) or the anti-inflammatory agent SASP (500 mg/kg) twice a day (once in the morning and once in the evening) by gavage with 0.5% CMC-Na solution as solvent. From day 1 to day 7, the mice were monitored daily for mental status, water consumption, body weight, fecal consistency, and fecal properties. During the modeling period, 1 mouse in the Pae-L group died.

At day 7 of the experiment, fresh mouse feces were collected and quickly frozen at −80 °C. The mice were fasted for 12 h before euthanasia. The colon length was measured, and the blood, liver, ileum, and colon tissues were quickly collected. A portion of ileal and colonic tissues was placed in 4% paraformaldehyde solution, another portion of colonic tissues was placed in 4% glutaraldehyde solution, and the rest was stored at −80 °C for subsequent analysis.

### 2.3. Disease Activity Index (DAI)

The DAI scoring in mice was performed using the following equation, according to the method of Bang et al. [37]: DAI = (weight loss score + stool trait score + blood in stool score)/3. Scoring details are shown in Table 1.

### 2.4. Histological Examination

Tissues of the distal colon of mice were fixed in 4% paraformaldehyde overnight, dehydrated in gradient ethanol, embedded in paraffin, sectioned, stained with hematoxylin and eosin and then observed under a microscope (Nikon Eclipse ci microscope).

### 2.5. Transmission Electron Microscopy (TEM)

The colon tissue fixed in 4% glutaraldehyde was sectioned, fixed, gradient dehydrated, embedded, cured, cut into thin sections, double stained with uranyl acetate and lead citrate, and placed under TEM for observation and photography.

### 2.6. ELISA Analysis

Inflammatory markers in mouse serum were determined using IL-4, IL-1β, IL-6 and TNF-α kits (Hangzhou Unitech Biotechnology Co., Ltd.), and all steps were performed strictly according to the manufacturer instructions. The absorbance was measured at 450 nm using an enzyme marker (Spectra Max i3x, Molecular Device, USA).

### 2.7. Immunohistochemistry

Immunohistochemical analysis was performed according to the reported method. Briefly, the paraffin-embedded distal colon was incubated with ZO-1 (1:100) and Occludin (1:200) primary antibodies, and the liver and ileum were incubated with FXR (1:200) and FGF15 (1:200) primary antibodies overnight at 4 °C, followed by 40 min incubation with biotinylated secondary antibodies. The signals were then visualized using the DAB concentration kit and hematoxylin re-staining.

### 2.8. RT-qPCR

Total RNA was extracted from ileal and liver tissues using an RNA Rapid Extraction Kit (AC0202, Sparkjade, Jinan, China) and measured using a NanoDrop 2000C spectrophotometer (Thermo Fisher Scientific, Waltham, MA, United States). Then, RT-qPCR was performed according to the direction of Fast SYBR Mixtrue (AH0104-B, Sparkjade, Jinan, China) and analyzed on an ABI 7500 FAST system. The 2(-ΔΔCt) method was used, with GADPH as an internal control. The primer sequences are provided in Table 2.

### 2.9. Western Blotting

Total colonic tissue proteins were extracted with radioimmunoprecipitation assay (RIPA) lysis buffer, and total mouse ileal terminal and liver tissue protein concentrations were determined by the BCA method. The following antibodies were used for protein blotting as described [38]: CYP7A1 (1:1000, Cat.#:861909; ZenBio), FXR (1:1000, Rb#bs-12867R; Bioss), FGF15 (1:1000, Mouse#sc-514647; Santa), and β-actin (1:1000, Mouse#AF0003; Beyotime). Mouse ileal terminal tissue was assayed for FXR and FGF15, and liver tissue was assayed for CYP7A1, FXR, and FGF15. Finally, the images were examined using the enhanced chemiluminescence (ECL) detection system (Bio-Rad, Richmond, CA, USA), and the grayscale of each band was quantified using ImageJ analysis software.

### 2.10. Gut Microbiota Profiling by 16S rRNA Sequencing

Total microbial genomic DNA was extracted from fecal samples using the PowerSoil DNA Isolation kit (Qiagen) according to manufacturer’s instructions. The quality of DNA extraction was verified using 1.0% agarose gel electrophoresis, and the DNA concentration and purity were further determined using NanoDrop2000. The hypervariable region of the 16S rRNA gene V3-V4 was PCR amplified using the primers 338F (5’-ACTCCTACGGGAGGCAGCAG-3’) and 806R (5’-GGACTACHVGGGTWTCTAAT-3’). PCR amplification cycling conditions were as follows: initial denaturation at 95 °C for 3 min, followed by 27 cycles of denaturation at 95 °C for 30 s, annealing at 55 °C for 30 s and extension at 72 °C for 45 s, a single extension at 72 °C for 10 min, and a final 4 °C step. Amplification products were purified using the QIAquick PCR purification kit (Qiagen), pooled in equimolar amounts, and paired-end sequenced on an Illumina MiSeq PE300 platform (Illumina, San Diego, USA).

### 2.11. Targeted Fecal Metabolomics Analysis

Fecal samples were thawed on ice. The metabolomics analysis was performed on a Q300 Kit (Metabo-Profile, Shanghai, China) according to the instructions of the manufacturer. Briefly, approximately 5 mg of each lyophilized sample was weighed and transferred to a new 1.5 mL tube with 25 μL of water. The samples were homogenized with zirconium oxide beads for 3 min, and 120 μL of methanol containing internal standard was extracted for gut metabolites. The samples were homogenized for another 3 min and then centrifuged at 18,000 rpm for 20 min. Then, 20 μL of supernatant was transferred to a 96-well plate with 20 μL of freshly prepared derivative reagents, and the derivatization was carried out at 30 °C for 60 min. After derivatization, 330 μL of ice-cold 50% methanol solution was added to dilute the sample. Then the plate was stored at −20 °C for 20 min, followed by centrifugation at 4,000 g at 4 °C for 30 min. A total of 135 μL of supernatant was transferred to a new 96-well for analysis.

All target standards were purchased from Sigma-Aldrich (St. Louis, MO, USA), Steraloids Inc. (Newport, RI, USA) and TRC Chemicals (Toronto, ON, Canada). Each sample or standard curve sample was loaded onto the ACQUITY UPLC BEH C18 1.7 µM VanGuard pre-column (2.1 × 5 mm) and ACQUITY UPLC BEH C18 1.7 µM analytical column (2.1 × 100 mm). The column temperature was 40 °C and the sample manager temperature was 10 °C. The mobile phase was water (A) plus 0.1% formic acid; acetonitrile (B); IPA (70:30). The following gradient elution procedure was used: 0–1 min (5% B), 1–5 min (5–30% B), 5–9 min (30–50% B), 9–11 min (50–78% B), 11–13.5 min (78–95% B), 13.5–14 min (95–100% B), 14–16 min (100% B), 16–16.1 min (100–5% B), 16.1–18 min (5% B), flow rate 0.40 mL/min, injection volume 5.0 uL. Raw data files generated by UPLC-MS/MS were processed using MassLynx software (v4.1, Waters, Milford, MA, USA) for peak integration, calibration and quantification for each metabolite. The self-developed platform iMAP (v1.0, Metabo-Profile, Shanghai, China) was used for statistical analyses, including PCA, PLS-DA, univariate analysis, and pathway analysis.

### 2.12. Statistical Analysis

Statistical analysis was performed using GraphPad Prism 7.04 (GraphPad, San Diego, CA). Data are presented as the mean ± SEM. One-way or two-way analysis of variance (ANOVA) followed by Tukey’s multiple comparison’s test was used to compare multiple groups. *p* < 0.05 was considered statistically significant.

## 3. Results

### 3.1. Pae Attenuates the Effect of DSS-Induced UC in Mice

**Figure 2:**
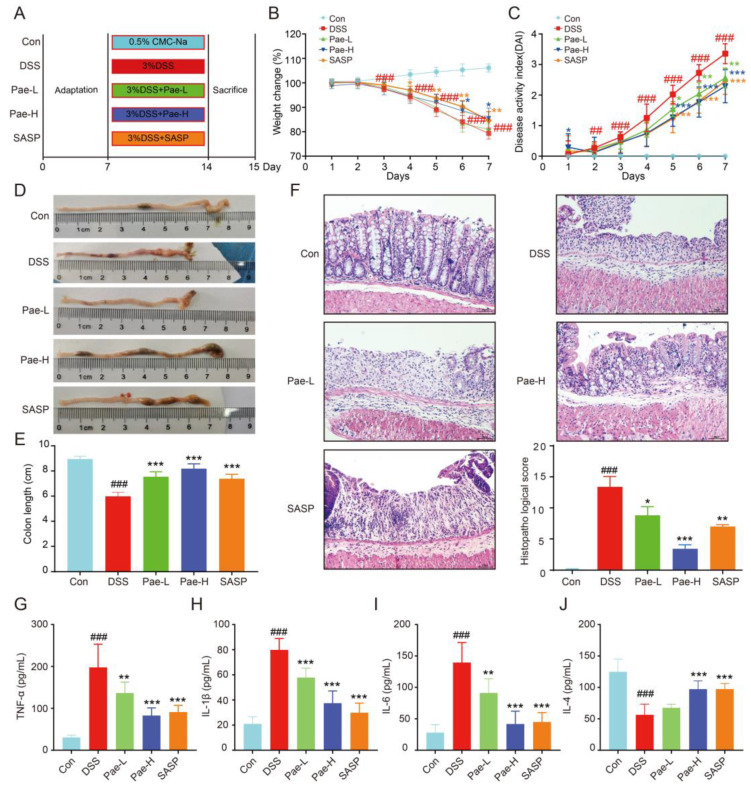
Pae attenuates the effect of DSS-induced UC in mice. (**A**) Experimental design. (**B**) Changes in body weight (*n* = 8). (**C**) Disease activity index (DAI) scores (*n* = 8). (**D,E**) Images of colon samples and changes in colon length (n = 7 or 8). (**F**) Representative images of hematoxylin and eosin (**E**,**H**)-stained colon tissue and the scores of (**E**,**H**), scale bar = 200 µm, (*n* = 5). (**G**–**J**) Expression levels of TNF-α (**G**) IL-1β (**H**), IL-6 (**I**) and IL-4 were detected by enzyme-linked immunosorbent assay (ELISA) (*n* = 6-8). Data are shown as mean ± sem. ## *p* < 0.01; ### *p* < 0.001, compared with Con group, * *p* < 0.5; ** *p* < 0.01; *** *p* < 0.001, compared with DSS group.

To confirm that Pae has an ameliorative effect on UC mice, we divided mice into five groups: Control (untreated, *n* = 8), Model (3% DSS, *n* = 8), Pae-L (3% DSS + 50 mg/kg Pae, *n* = 7, Pae-H (3% DSS + 100 mg/kg Pae, *n* = 8) and SASP (3% DSS + SASP, *n* = 8). The mice were treated for one week (Figure 2A), and throughout the treatment period, the body weight and DAI were recorded as indicators of the success of UC model construction. As expected, DSS caused a weight decrease (starting at day 3) and a rise in the DAI score (starting at day 2). Furthermore, these changes were decreased by the anti-inflammatory agent SASP, as expected. The DAI and weight changes were also suppressed by Pae-H, although the Pae-L group did not significantly inhibit body weight loss (Figure 2B,C). Consistently, the colon length was significantly shorter in the DSS group than in the Control group, but this effect was significantly suppressed in the Pae-L, Pae-H and SASP groups (Figure 2D,E).

A primary pathological feature of UC is the expansion of intestinal inflammation and subsequent disruption of epithelial barrier function. As expected, histopathological analysis revealed no obvious tissue pathological morphology for the Control group. However, for the DSS group, severe defects were observed in the upper colonic mucosa, and DSS also caused crypt damage, localized erosions and ulcers, and inflammatory cell infiltration, which were improved to different degrees by the administration of Pae-L, Pae-H and SASP (Figure 2F).

To further verify the effect of Pae on inflammation in UC mice, we evaluated the levels of inflammatory cytokines by ELISA. The levels of IL-6, TNF-α and IL-1β pro-inflammatory cytokines in the serum of mice from the DSS group as compared to the Control group were significantly increased, while the anti-inflammatory factor IL-4 was significantly decreased. Furthermore, Pae-L, Pae-H and SASP significantly reversed these trends, with the exception that the effect of Pae-L on IL-4 was not significant (Figure 2G–J). Taken together, these results emphasize the palliative effect of Pae, and especially Pae-H, in UC mice.

### 3.2. Pae Restores Intestinal Barrier Function in DSS-Induced UC

**Figure 3:**
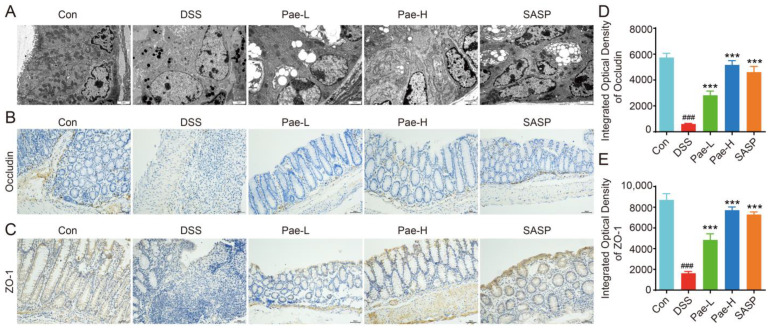
Pae restores intestinal barrier function in DSS-induced UC mice. (**A**) Representative images of colon tissue observed under a transmission electron microscopy (×10,000) (*n* = 3). (**B**,**C**) Immunohistochemistry staining (scale bar, 50 mm) (*n* = 5). (**D**,**E**) quantitative analysis of Occludin and ZO-1 protein content in the colon (*n* = 5). Data are shown as mean ± sem. ### *p* < 0.001, compared with Con group, *** *p* < 0.001, compared with DSS group.

Inflammatory cytokine overexpression, which disrupts intestinal function, leads to alterations in tight junction (TJ) proteins in the intestinal epithelial barrier [39]. To assess the protective effect of Pae on colonic epithelial mucosal integrity in UC mice, we performed TEM. Compared with the Control group, the TJs between colonic epithelial tissues in the DSS group were loosened, blurred, and less dense, and the bridging granular structures were lost. However, the Pae-L, Pae-H and SASP groups showed different degrees of improvement in the TJs, with clear tight links, narrowed cell gaps and smaller organelles for the Pae-H group (Figure 3A).

We also performed immunohistochemistry to detect the effect on Occludin and ZO-1 (Figure 2B,C), which are associated with tight linkage. Compared with the Control group, the expression levels of these proteins were significantly lower in the DSS group; while compared with the DSS group, their expression was significantly higher in the Pae-L, Pae-H, and SASP groups (Figure 3D,E). The treatment effect was best for the Pae-H group, which is consistent with the observation of TEM. Therefore, these results support the protective effect of Pae on the TJs in UC mice.

### 3.3. Pae Attenuates DSS-Induced Dysregulation of the Gut Microbiota in Mice with UC

**Figure 4:**
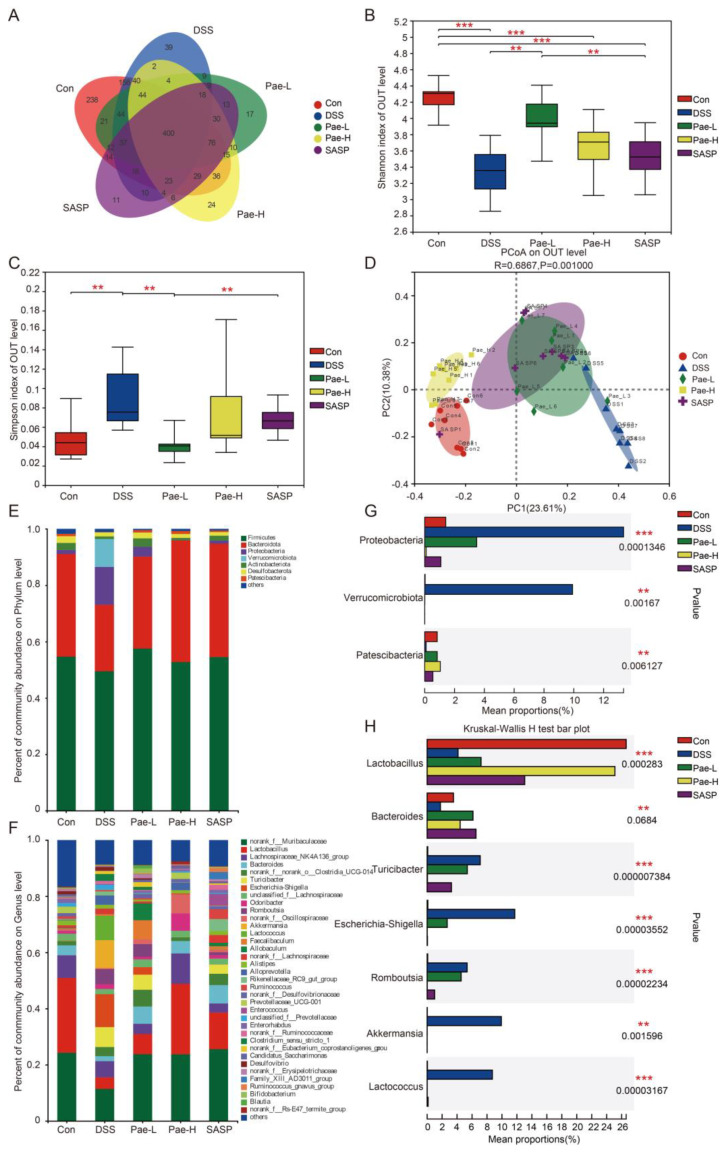
Effects of Pae on the fecal gut microbiota alterations in DSS-induced UC mice. (**A**) Venn diagram showing common species comparison with the five groups. (**B**) The Shannon index of the gut microbiota. (**C**) The Simpson index of the gut microbiota. (**D**) Multiple sample PCoA of the Bray-Curtis distance based on OTUs. (**E**) Microbial community bar plot at phylum level. (**F**) Relative abundance of the gut microbiota at the genus level. (**G**,**H**) Relative abundance of the significantly altered bacteria at the family and genus levels from the five groups. Data are presented as the mean ± sem. *n* = 7 or 8. ** *p* < 0.01; *** *p* < 0.001.

Next, we evaluated the fecal microorganisms of mice by 16S rDNA amplicon sequencing to explore the alleviating effect of Pae on DSS-induced UC. Among 39 samples, 1400 operational taxonomic units (OTUs) were obtained, of which 400 were common to the five groups. There were 238, 39, 17, 24, and 11 OTUs specific to the Control, DSS, Pae-L, Pae-H, and SASP groups, respectively (Figure 4A). Furthermore, the abundance and diversity of the intestinal microbiota in the DSS group were significantly less than those in the Control group, while this reduction was less apparent after Pae or SASP administration (Figure 4B,C). These results indicate that Pae partially reversed the dysregulation of the intestinal microbiota in UC mice. Consistent with these findings, principal coordinate analysis of the Bray-Curtis distances also showed a significant separation between the Control and DSS groups. Interestingly, the OTU of mice treated with Pae or SASP showed a trend of separation with regional crossover, but the Pae-H group was completely separate from the DSS group and close to the Control group (Figure 4D).

Further analysis of the taxonomic distributions showed differences in microbial composition between the groups of mice (Figure 4E,F, Supplementary Tables S1 and S2). At the phylum level, Firmicutes and Bacteroidota accounted for the largest proportion of phyla, which is consistent with previous findings [40,41]. The relative abundance of Patescibacteria was significantly lower in the DSS group than in the Control group, while the relative abundance of Verrucomicrobiota was significantly higher and the abundance of Proteobacteria showed a trend of being higher. In both the Pae-L and Pae-H groups, there was significantly higher Patescibacteria relative to the abundance in the DSS group; and the Pae-H group displayed lower abundance of Proteobacteria and Verrucomicrobiota relative to the abundance in all administration groups (Figure 4G) At the genus level, the DSS group had significantly lower abundance compared to the levels in the Control group of BA-related bacteria, including the probiotic Lactobacillus, Bacteroides spp., while Pae-H (but not SASP and Pae-L) significantly increased the abundance of these species (Figure 4H). In addition, relative to the Control group, the DSS group displayed significantly increased abundance of Turicibacter, Romboutsia Akkermansia, Lactococcus and the harmful bacteria Escherichia coli-Shigella. Interestingly, Pae-L, Pae-H and SASP each restored the DSS-induced intestinal gut microbiota imbalances in Akkermansia and Lactococcus; while SASP also significantly reversed the UC-associated increase in Romboutsia, and Pae-H also significantly reversed the UC-associated increases in Turicibacter, Bacteroides, and Romboutsia and promoted a trend of decrease in Escherichia-Shigella. These results suggest that Pae can increase the proportion of probiotic bacteria, reduce pathogenic bacteria, enhance intestinal barrier function and promote the recovery of the gut microbiota.

### 3.4. Effect of Pae on Fecal Metabolic Disorders Caused by DSS-Induced UC in Mice

**Figure 5:**
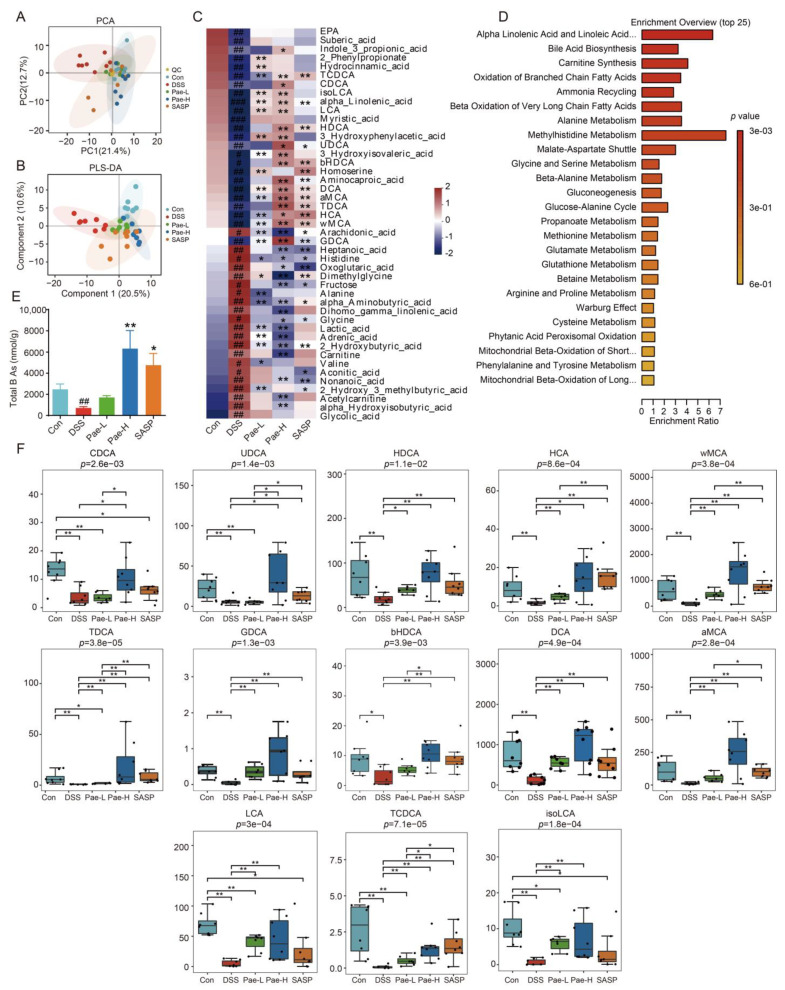
Effects of Pae on fecal metabolism in DSS-induced UC mice. (**A**) Principal Component Analysis (PCA) score plot of Con, DSS, Pae-L, Pae-H and SASP groups and QC samples. (**B**) Partial Least Squares Discriminant Analysis (PLS-DA) score plot from Con, DSS, Pae-L, Pae-H and SASP groups. (**C**) Heatmap of fecal differential metabolite profiles in mice. (**D**) Metabolic pathway enrichment analysis. (**E**) Total BA concentrations in fecal contents. (**F**) Quantitative abundance of significantly altered BAs from different groups (nmol/g). Date are shown as mean ± sem. *n* = 7 or 8.# *p* < 0.5; ## *p* < 0.01; ### *p* < 0.001, compared with Con group, * *p* < 0.5; ** *p* < 0.01, compared with DSS group.

Gut microbiota actions are closely linked to the host microbial metabolic axis, for which metabolomics is a useful tool to reveal the interactions between the host and the gut microbiota. Thus, we further investigated the fecal metabolism of DSS-treated UC mice using UPLC-MS/MS. A total of 163 metabolites were identified and quantified, including amino acids, short-chain fatty acids, BAs, fatty acids, carbohydrates, organic acids, benzenoids, pyridines, phenols, phenylpropanoic acids, benzoic acids, indoles, phenylpropanoids and carnitines. In PCA analysis, the quality control sample points were close to each other and aggregated to a high degree, suggesting good stability of the instrumental assay (Figure 5A). Furthermore, in partial least squares-discriminant analysis, there was clear separation between the control and DSS groups, while the Pae treatment group was distant from both the Control and DSS groups (Figure 5B), which supports the possibility that Pae regulates the metabolism of the intestinal microbiota.

Based on the criteria of *p* < 0.05,|log2(FC)| > 1 and VIP > 1 for screening, 45 metabolites were significantly changed in the DSS group compared with the Control group, with 25, 24, and 34 metabolites significantly reversed after SASP, Pae-L, and Pae-H treatment, respectively (Figure 5C, Supplementary Table S3). Using the MetaboAnalyst online website, the top three metabolic pathways in the Control versus DSS groups were “Alpha Linolenic Acid and Linoleic Acid Metabolism”, “Bile Acid Biosynthesis”, and “Alanine Metabolism” (Figure S1). Furthermore, Pae-H significantly reversed the DSS-induced changes in “Linolenic Acid and Linoleic Acid Metabolism” and “Bile Acid Biosynthesis” (Figure 5D), Pae-L significantly reversed the DSS-induced changes in “Alpha Linolenic Acid and Linoleic Acid Metabolism” (Figure S2), and SASP significantly reversed the DSS-induced changes in “Bile Acid Biosynthesis” and “Ammonia Recycling” (Figure S3).

The gut microbiota is thought to play important roles in the development of UC by regulating the pool size and composition and altering the chemical and signaling properties of BAs [22,42,43]. Therefore, we identified and quantified 27 BAs that were significantly lower in the DSS group compared to the Control group. Furthermore, the decrease in the levels of the BAs was significantly reversed after the administration of Pae-L, Pae-H and SASP (Figure 5E). Specifically, the levels of 13 BAs metabolites were significantly lower in the DSS group compared to the Control group and also were significantly higher in the Pae-H group compared to the DSS group (Figure 5F). This includes ligands related to the nuclear receptor FXR of BAs, including CDCA, LCA, and DCA. Thus, Pae intervention of BA metabolism via modulation of the gut microbiota may contribute to its ability to alleviate UC in the DSS mouse model.

### 3.5. Pae Improves UC via Pathways Involving Gut Microbiota-BAs-FXR/FGF15 Signaling

As intestinal flora is strongly associated with bile acids and is closely related to the development of colitis, we explored the relationship between Pae regulation of altered gut microbiota and BAs homeostasis in UC mice, and tentatively validated the FXR/FGF15 pathway associated with BAs.

**Figure 6:**
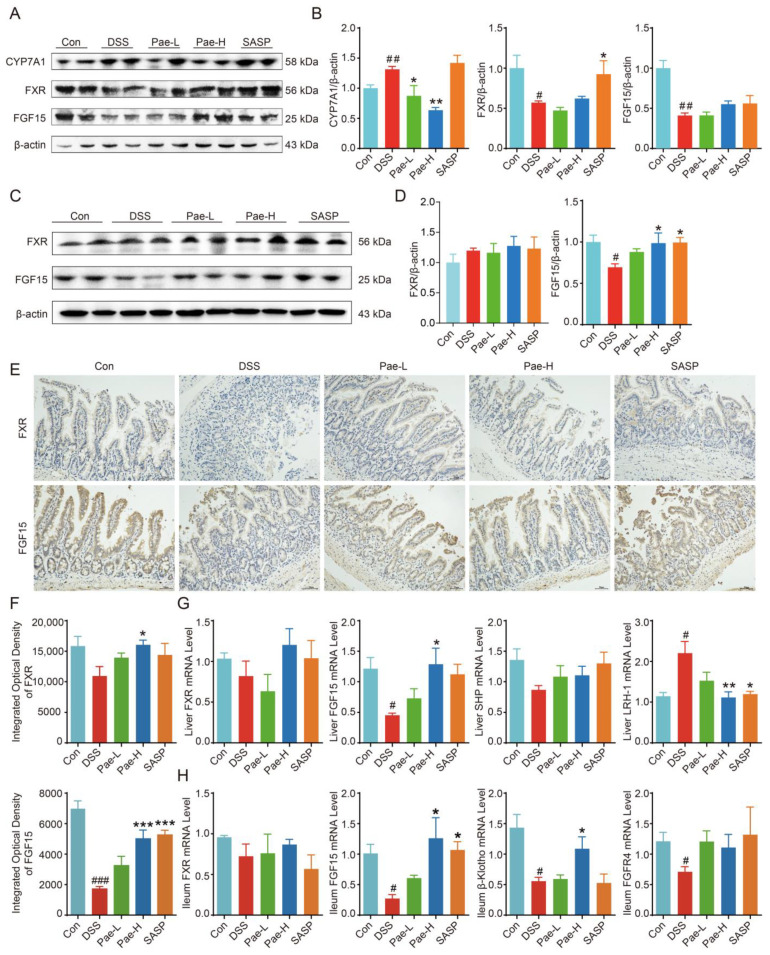
Pae improves UC via pathways involving gut microbiota-BAs-FXR/FGF15 signaling. (**A**,**B**) Representative immunoblots and the relative expression levels of CYP7A1, FXR and FGF15 proteins in liver tissues from different treatment groups. (**C**,**D**) Representative immunoblots and the relative expression levels of FXR and FGF15 proteins in ileum tissues from different treatment groups. (**E**,**F**) Immunohistochemistry staining and quantitative analysis of FXR and FGF15 protein content in the distal ileum (scale bar, 50 mm). (**G**) The mRNA levels of FXR, FGF15, SHP and LRH-1 for each group in the liver. (**H**) The mRNA levels of FXR, FGF15, β-Klotho and FGFR4 for each group in the ileum. Data are presented as the mean ± sem. *n* =3 or 4. # *p* < 0.5; ## *p* < 0.01; ### *p* < 0.001, compared with Con group, * *p* < 0.5; ** *p* < 0.01; *** *p* < 0.001, compared with DSS group.

Compared with the expression in the Control group, CYP7A1 protein expression was significantly higher and FXR and FGF15 expression was significantly lower in the livers of the DSS group of mice (Figure 6A,B). After administration of Pae-L and Pae-H, CYP7A1 protein expression was significantly reduced, while the effect on FXR and FGF15 expression was not significant; and after administration of SASP, FXR protein expression was significantly higher, while the effect on CYP7A1 and FGF15 expression was not significant. We also evaluated the expression of FXR and FGF15 proteins in the ileum (Figure 6C). Compared to expression in the Control group, the expression of FXR was not statistically different in the DSS group of mice, and the expression of FGF15 was significantly lower. After the administration of Pae and SASP, the expression of FGF15 was significantly upregulated in the Pae-H and SASP groups (*p* < 0.05), while there was no statistical difference in the Pae-L group (Figure 6D). In immunohistochemical analysis of the ileum (Figure 6E), FGF15 expression was significantly lower in the DSS group compared with the Control group, whereas there was no statistical difference in FXR expression. Additionally, FGF15 expression was significantly upregulated in the Pae-H and SASP groups, and FXR expression was significantly upregulated in the Pae-H group (Figure 6F).

To further verify FXR-FGF15 signaling changes in the liver and ileum of UC mice, we examined effects on mRNA expression. In the liver, there was no significant difference in FXR and SHP mRNA expression, though the mRNA expression of LRH-1 was significantly higher and the mRNA expression of FGF15 was significantly lower in the DSS group as compared to the Control group. Furthermore, the expression changes of FGF15 and LRH-1 were reversed after Pae-H administration, and the expression change of LRH-1 was reversed after SASP administration (Figure 6G). In ileum tissue, FXR was not significantly changed in the DSS group compared with the Control group. However, mRNA expression of FXR target genes, including Fgf15, β-klotho and FGFR4, was significantly decreased in the DSS group, while after Pae-H intervention, FGF15 and β-klotho were significantly increased, and after SASP intervention, FGF15 was significantly increased (Figure 6H). Altogether, these data suggest that Pae may influence BA synthesis by modulating liver FXR-SHP/LRH-1 and ileum FXR-FGF15 signaling pathways, thereby improving UC in mice.

### 3.6. Integrated Map of the Mechanism of Pae in Ameliorating UC

**Figure 7:**
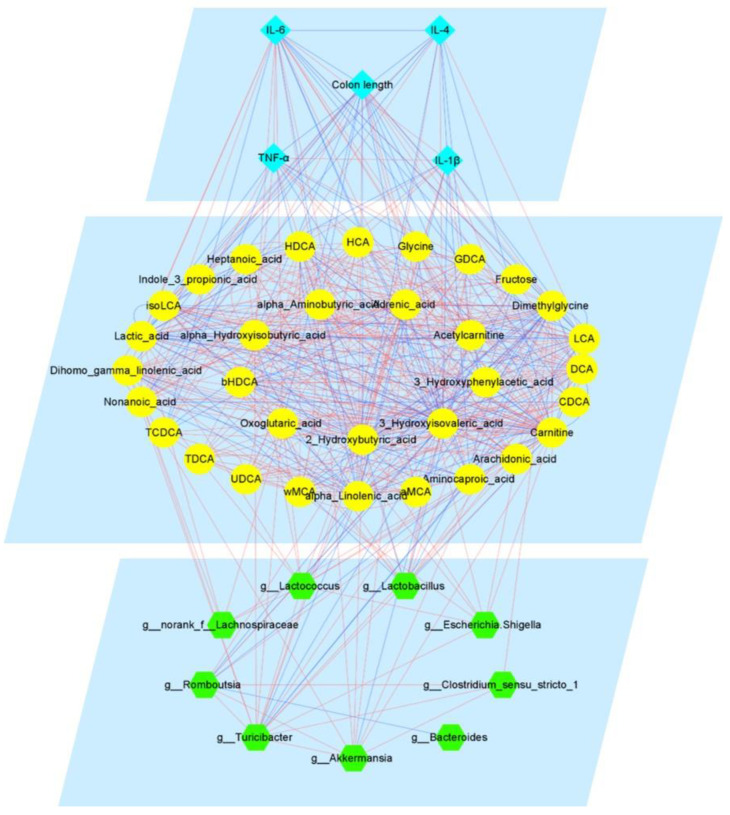
The integrated therapeutic mechanism of Pae in treating UC. The edges are the correlations with Spearman’s correlation coefficient < −0.6 (or >0.6) and *p* < 0.05. The inner layer edges are represented by blue lines and the between layer edges are represented by red lines.

To comprehensively evaluate our data and support a system-level understanding of disease and drug mechanisms, we employed an integrative analysis of data from three dimensions: genus-level gut microbiota, fecal metabolites, and phenotypic data [44]. Specifically, we chose the Control, DSS and Pae-H groups for association analysis, given that the treatment effect of the Pae-H group appeared to be superior to that of the Pae-L and SASP groups. To start with, we evaluated intra-layer correlations, including correlations between the gut microbiota, fecal metabolites and phenotypes; the gut microbiota and fecal metabolites; and fecal metabolites and phenotypes. Data with correlation coefficients greater than 0.6 were used to construct an integrated visualization network containing three layers. As shown in Figure 7, nine taxa in the gut microbial layer, including Lactococcus, Lactobacillus, Akkermansia and Bacteroides, and 33 metabolites in the fecal metabolite layer, including LCA, CDCA, DCA, isoLCA, Acetylcarnitine and Glycine, were integrated into the network. In the phenotypic layer, five phenotypic parameters, including colon length, IL-4, IL-6, IL-1β, and TNF-α, were integrated. The results demonstrate that the levels of six BAs, including LCA, isoLCA, DCA, HDCA, bHDCA, and GDCA, were positively correlated with the abundance of Lactobacillus. Furthermore, LCA, TCDCA, isoLCA, DCA, and CDCA were positively correlated with colon length; LCA, TCDCA, and isoLCA were positively correlated with IL-4; LCA, TCDCA, DCA, and CDCA were negatively correlated with IL-6; LCA and isoLCA were negatively correlated with IL-1β; and LCA, TCDCA, isoLCA, and CDCA were negatively correlated with TNF-α. The comprehensive network provides us with a holistic view of the therapeutic mechanism of Pae for UC.

## 4. Discussion

UC is a clinically common inflammatory bowel disease characterized by intestinal inflammation, bloody diarrhea, and abdominal pain, requiring long-term effective pharmacological treatment [45]. Pae is a natural phenolic compound widely found in the root bark of the peony flower, a medicinal and food plant that has been found to show potential in the treatment of UC [33]. Recent results of the latter study show that clinical symptoms of UC in mice can be significantly improved after Pae intervention, which encouraged us to further investigate the mechanism by which Pae ameliorates UC. In this study, we confirmed the therapeutic effect of Pae on UC and further demonstrated that its mechanism is closely related to the inflammatory status of the colon, as well as the intestinal microbiota and metabolites. Our findings enabled us to construct a comprehensive network to synthesize the understanding of several interrelated Pae mechanisms in improving UC in the DSS mouse model.

One measure of the extent and prognosis of UC is the DAI, which is calculated based on disease signs and symptoms and is usually considered a standard index of UC [46]. We verified that DSS significantly increased the DAI. Furthermore, mice in the Pae group had increased body weight, longer colonic length, thicker fecal viscosity and less blood in the feces compared to mice the DSS group, which resulted in a significantly lower DAI in the Pae group. In histopathological assessments of colonic tissue, Pae alleviated upper colonic mucosal defects, crypt damage, local ulceration and inflammatory cell infiltration in UC mice, thereby restoring intestinal epithelial mucosal integrity. Therefore, these results verify the therapeutic potential of Pae.

There is growing evidence of a role for intestinal epithelial barrier disruption and inflammation in UC. Changes in TJ proteins lead to disruption of the intestinal epithelial barrier, which allows microorganisms in the intestinal lumen to promote abnormal immune responses, with excessive leakage of bacterial antigens from the mucosa gradually degrading the TJs [47,48]. TNF-α impairs the intestinal barrier by inducing apoptosis in epithelial cells and altering the structure and function of the TJ [49], while IL-1β has been shown to increase TJ permeability in human intestinal epithelial cells [50]. In Caco-2 cells, IL-6 regulates TJ permeability through PI3K and MEK/ERK pathways, and the intestinal mucosa of IL-6-overexpressing mice exhibits increased expression of TJ proteins, which negatively correlates with cellular by-pass [51]. On the other hand, IL-4 has been shown to reduce the epithelial barrier function of a T84 cell monolayer [52]. Therefore, re-establishment of the integrity of the intestinal barrier may be a beneficial strategy for mediating the inhibition of inflammation in UC. In our study, we confirmed that oral administration of Pae (50 or 100 mg/kg) decreased the expression of inflammatory cytokines and increased the expression of the TJ proteins ZO-1 and Occludin, suggesting a mechanism by which Pae restores the structure and function of the epithelial barrier to alleviate the effects of UC.

Many previous studies have shown that the gut microbiota plays a key role in the pathogenesis of UC, with changes in the gut microbiota associated with repair of the intestinal mucosa, improvement in the inflammatory response, and enhanced immunity, leading to remission or even a cure for the disease [53,54]. In the present study, we demonstrated that UC mice treated with either Pae or SASP showed a significant change in intestinal microbial composition, suggesting that the protective effect of Pae on DSS-induced UC may be related to the regulation of the gut microbiota. These observations are consistent with previous reports demonstrating that reprogramming the balance of the intestinal microbiota improves UC [40]. Notably, Pae treatment significantly increased the abundance of Lactobacillus and Bacteroides. Specifically, Lactobacillus and Bacteroides are known to enhance intestinal barrier function by promoting the expression of intestinal epithelial cell TJ proteins, reducing apoptosis of epithelial cells and regulating the thickness of the intestinal mucus layer [55,56,57]. The latter reports support the possibility that Pae may reduce DSS-induced intestinal inflammation in UC mice by increasing the abundance of Lactobacillus and Bacteroides. We also observed significant elevation of pathogenic bacteria such as Escherichia-Shigella upon DSS-induced UC, which is consistent with the results of another study [58,59]. Escherichia-Shigella are typical genera of Aspergillus that includes LPS-producing Gram-negative bacteria and are thought to cause an imbalance in Th17/Treg conversion, leading to disruption of intestinal immune homeostasis [60,61]. Other studies have indicated that Escherichia-Shigella coli adherence to colonic mucosal epithelial cells positively correlates with pro-inflammatory cytokines, leading to high expression of inflammatory cytokines and thus disrupting the integrity of the intestinal barrier [60]. Therefore, the increase in potential beneficial bacteria and decrease in pathogenic bacteria observed in our study suggests that Pae may restore the intestinal microbiota to maintain the stability and function of the internal environment.

Evidence suggests that alterations in the composition of gut microbiota may modulate metabolic pathways, which impacts energy and mucosal immune homeostasis [62]. Therefore, we analyzed fecal metabolite profiles to further understand the host microbiota interactions associated with UC. In this context, our results show that the levels of BAs, organic acids and fatty acids were significantly reduced in DSS-induced UC mice, and alterations in selected metabolites were significantly reversed after Pae treatment. In particular, the concentrations of the secondary BAs DCA, LCA and CDCA in the feces of UC mice were significantly increased after Pae treatment; however, changes in the concentrations of primary BAs CA were not significant, which could be explained by individual differences in mice or the complexity of their metabolites. Nevertheless, the general trend of this change is consistent with the well-characterized role of BAs in UC, with evidence for a potential role for Pae in reversing their production.

Previous studies have also shown that deletion of unbound and secondary BAs leads to activation of the farnesol X receptor (FXR), which may impair anti-inflammatory pathways and intestinal barrier function, ultimately contributing to the pathogenesis of inflammatory bowel disease [11,63]. Additionally, DSS-induced UC has been shown to promote the accumulation of BAs in the colon by inhibiting the activation of the FXR-FGF15 signaling pathway [64]. FXR in the ileum has also previously been shown to activate the expression of FGF15, which enters enterohepatic circulation, binds to β-KLOTHO-mediated FGFR4 and downregulates the expression of CYP7A1, thus inhibiting the synthesis of BAs [65,66]. In our recent study, we suggest that the mechanism by which Pae regulates the metabolic pathway of BAs in UC may be through the regulation of hepatic FXR-SHP/LRH-1 and ileal FXR-FGF15 pathways, which in turn affects the expression of CYP7A1, thereby enhancing the synthesis of BAs. Consistently, in this study we demonstrated that in the liver, the expression of FXR and FGF15 trended downward in the DSS group of mice and upward after the administration of Pae. Pae intervention also increased the expression of FXR mRNA, FGF15 mRNA and SHP mRNA, and down-regulated LRH-1 mRNA expression. In the ileum, Pae trended to or significantly reversed the DSS-induced changes in the expression of FXR and FGF15, leading to the inhibition of CYP7A1 expression. We further demonstrated that FGF15, β-KLOTHO and FGFR4 mRNA levels in the ileum were significantly decreased in the DSS group. With Pae intervention, FGF15 and β-KLOTHO mRNA levels were significantly increased, and FXR and FGFR4 mRNA expression had a trend of increase, indicating that the synthesis of BAs in ileal tissues might be inhibited after Pae administration.

To gain a comprehensive understanding of the mechanisms of Pae in improving UC, we constructed an association network using the Spearman’s hierarchical correlation method, with data regarding the genus-level gut microbiota, fecal metabolites, and phenotype of UC mice. Interestingly, the abundance of Lactobacilli correlated with increased levels of LCA and CDCA, which have been used to establish therapeutic effects on colitis in animal models [67,68]. Lactobacillus is the main bacterial genus involved in BA uncoupling and converts uncoupled primary BAs (e.g., CDCA) to secondary BAs (e.g., LCA) via CYP7A1-mediated 7α-dehydroxylation [64], suggesting that Pae may indirectly increase fecal secondary BA (e.g., LCA) levels by promoting the growth of related intestinal flora (e.g., Lactobacillus). Additionally, elevated BA levels were closely associated with inflammatory responses. BAs can directly act on epithelial cells to regulate the production of proinflammatory cytokines such as TNF-α, IL-1β, and IL-6, which suggests that BAs may in turn improve the disruption of intestinal microbiota through the regulation of inflammatory cytokines and thus improve the intestinal function of UC [69,70]. Taken together, the three-dimensional association network suggests that Pae may be useful in treating UC by modulating the composition of BAs, which in turn improves intestinal barrier function and microbial composition by increasing the proportion of probiotic bacteria and reducing pathogenic bacteria, thereby reducing clinical intestinal inflammatory symptoms in UC mice.

In summary, our study supports findings suggesting that Pae significantly improves colonic injury caused by UC and demonstrates that Pae protects intestinal mucosal integrity by restoring gut microbiota dysbiosis and regulating metabolic disorders to prevent DSS-induced UC symptoms.

## 5. Conclusions

In conclusion, our study demonstrates for the first time that Pae reverses DSS-induced UC in mice by interfering with gut microbes and fecal metabolites. This study demonstrates that Pae treatment increases the abundance of Lactobacillus in the feces of UC mice, which indirectly activates hepatic FXR-SHP/LRH-1 and ileal FXR-FGF15 pathways associated with the synthesis of BAs to intervene with the expression of CYP7A1, the rate-limiting enzyme for BAs, thus restoring the metabolism of fecal BAs (e.g., DCA, LCA vs. CDCA) and ultimately ameliorating DSS-induced disruption of intestinal barrier function and colonic inflammation. In short, our findings provide a new biochemical mechanism of Pae against UC, which serves as a foundation for developing Pae as a clinical agent for treating UC.

## Figures and Tables

**Table 1:** The DAI scoring standards.

Body Weight Loss	Stool Consistency	Presence of Gross Bleeding or Bloodstain	Score
No loss	Normal	Negative	0
1–5%			1
5–10%	Loose stools	Positive	2
10–15%			3
Over 15%	Diarrhea	Gross rectal bleeding	4

**Table 2:** Primer sequences of the genes used for qRT-PCR.

Gene	Forward Primer	Reverse Primer
FGF15	ACCAGAAACCCTCAAACT	CTACATCCTCCACCATCC
FXR	CCATTTACAGGCTACGGA	ACTTGAGGAAACGGGACA
SHP	CCCAGCAAGGACACTGAGCAAG	CCTCGAAGGTCACAGCAT
LRH-1	CTGAGTCAATGATGGGTTA	CTTTTCTTGCCTGTTTCG
β-KLOTHO	ACCATTTGCTCATTTCTCG	ACTCTGCTGTGGCCTTTC
FGFR4	TGGGCTAATGAGGGAGTG	AGGCGGAGGTCAAGGTAC
GAPDH	CCCAGCAAGGACACTGAGCAAG	GGTCTGGGATGGAAATTGTGAGGG

## Data Availability

The 16S rDNA data can be found in the NCBI database (SRA data: PRJNA876396), and the metabolite data can be found in the MetaboLights database (MTBLS5204).

## Supplementary Materials

The following are available online at https://www.mdpi.com/article/10.3390/metabo12100956/s1, Table S1: otu taxon Phylum percent, Table S2: otu taxon Genus percent, Table S3: 45 differential metabolites,Figure S1: metabolic pathways in the control group compared with the model group, Figure S2: metabolic pathways in the Pae-L group compared with the model group, Figure S3: metabolic pathways in the SASP group compared with the model group.
