# Supplementary figures and images for: Paeonol Ameliorates Ulcerative Colitis in Mice by Modulating the Gut Microbiota and Metabolites
Source: Metabolites. 2022 Oct 8;12(10):956. doi: 10.3390/metabo12100956 (PMC9612301; doi:10.3390/metabo12100956)

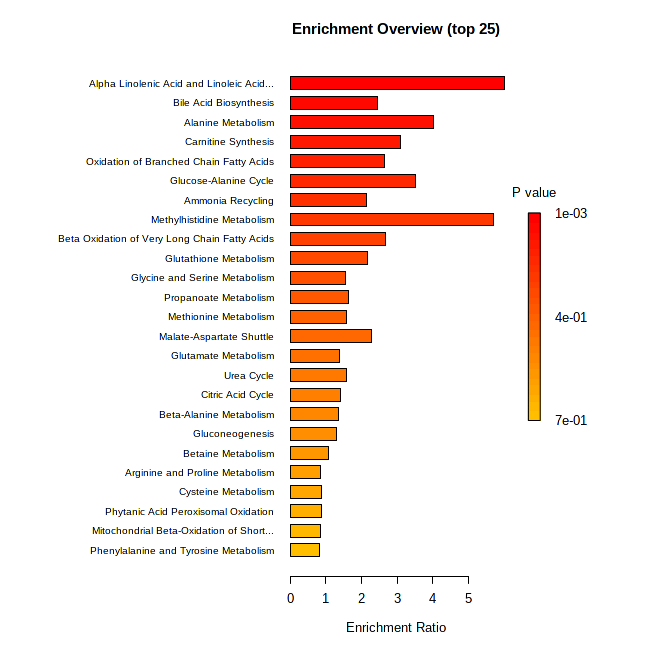

Supplement: Supplementary file 1 [file metabolites-12-00956-s001.zip › Figure S1.jpg]

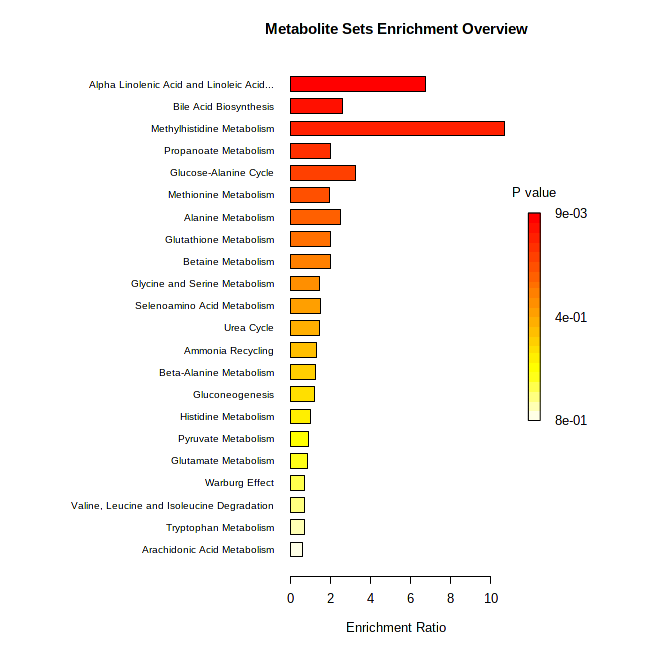

Supplement: Supplementary file 1 [file metabolites-12-00956-s001.zip › Figure S2.jpg]

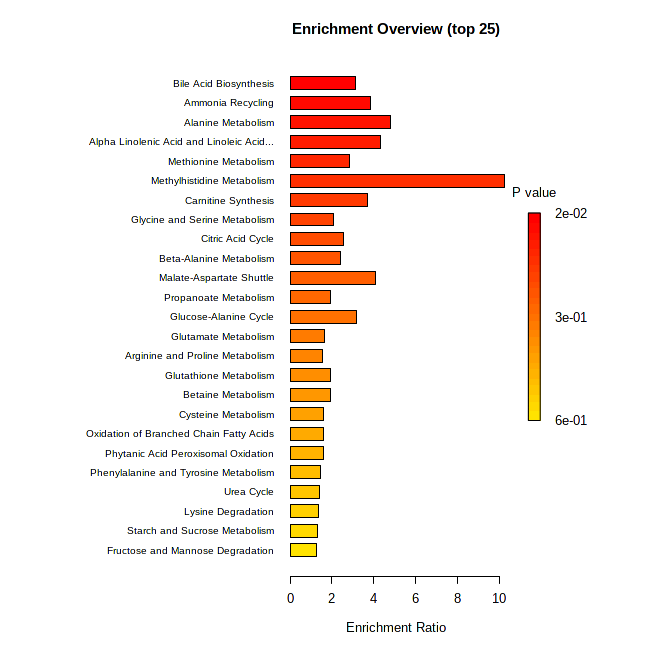

Supplement: Supplementary file 1 [file metabolites-12-00956-s001.zip › Figure S3.jpg]
